# Effect of ascorbic acid and epidermal growth factor in a rat tibia defect

**DOI:** 10.1590/acb381623

**Published:** 2023-05-01

**Authors:** Victor Manuel Domínguez-Hernández, Cecília Hernández-Flores, Alfonso Delgado, Rene Valdez-Mijares, Victor M. Araujo-Monsalvo, Olivia Hernández-González

**Affiliations:** 1 Instituto Nacional de Rehabilitación LGII – Laboratorio de Biomecánica – Ciudad de México, México.; 2 Instituto Nacional de Rehabilitación LGII – Laboratorio de Bioquímica – Ciudad de México, México.; 3 Universidad Autónoma de Chihuahua – Facultad de Medicina y Ciencias Biomédicas – Departamento de Fisiología – Chihuahua, México.; 4 Instituto Nacional de Rehabilitación LGII – Laboratorio de Microscopía Electrónica – Ciudad de México, México.

**Keywords:** Biocompatible Materials, Biomechanical Phenomena, Orthopedic Procedures

## Abstract

**Purpose:**

Bone repair aims to restore the anatomical, biomechanical, and functional integrity of the affected structure. Here we study the effects of ascorbic acid (AA) and epidermal growth factor (EGF) applied in a single dose and in combination on the repair of a noncritical bone defect model.

**Methods:**

Twenty-four rats were divided into four groups: an intact G-1 control group, and three groups that underwent a noncritical bone defect in the right tibia: G-2 treated with AA, G-3 treated with EGF, and G-4 treated with AA in combination with EGF. After 21 days of treatment, rats were sacrificed, the tibias were dissected and a destructive biomechanical analysis of three-point flexion test was performed in a universal testing machine; the values of stiffness, resistance, maximum energy, and energy at maximum load were statistically compared.

**Results:**

G-3 and G-4 recovered the biomechanical properties of strength and stiffness of an intact tibia 3 weeks after their application. Not so the energy and energy at maximum load. For G-2, only the stiffness of an intact tibia was recovered.

**Conclusions:**

EGF and AA-EGF applied to a noncritical bone defect in the rat tibia favors the recovery of bone resistance and stiffness.

## Introduction

The loss of a bone fragment (bone defect) can be attributed to different clinical conditions, such as trauma, surgical removal, oncological sequelae, or bone pathology. Recovery implies a cost for society, as well as for the health system and the patient. On some occasions, they can lead to long treatment periods, which depend on the location of the defect, the size of the defect and the patient’s state of health^1^^-^ 4.

There are different techniques to stimulate the repair of bone defects such as the application of grafts, application of growth factors, electrical stimulation, mechanical stimulation, as well as various types of scaffolding and combinations of scaffolding with cells, among others. Bone repair aims to restore the anatomical, biomechanical, and functional integrity of the affected bone^4^^–^ 7.

Bone repair is a complex process involving numerous molecular signaling cascades, specific cell types (osteoblasts and osteoclasts), molecular scaffolding (extracellular matrix molecules [collagen, fibronectin, hydroxyapatite]), molecular soluble (growth factors [TFG-β, EGF, FGF, VEGF], vitamins [C, K, D], cytokines [IL-1, IL-6, TNF-α], ions [Ca^2+^, P^3–^, Mg^2+^], hormones [parathyroid, calcitonin, insulin]). These factors form a microenvironment during the repair process that determines the efficacy of both fracture repair^8^ and bone defect repair^9^^,^ 10.

Growth factors are found in all tissues, they are important for the repair process to be successful. They are known to stimulate cell proliferation, migration, and differentiation^11^. They have been used in various models to investigate their effect, applying them both endogenously and exogenously^12^^,^ 13. Some growth factors act during bone repair, such as epidermal growth factor (EGF), which is known to stimulate osteoblast proliferation, development, and growth, as well as extracellular matrix mineralization^2^. On the other hand, EGF can stimulate bone resorption in rat and human long bone culture, regulating osteoclastogenesis as well as the balance in bone turnover, this can also be achieved through synergistic effects with other growth factors^11^.

In *in vitro* and *in vivo* studies, growth factors have been documented to have multiple regulatory effects on bone cells, induce bone repair, proliferation, differentiation, and metabolism^14^. In addition, growth factors such as EGF, fibroblast growth factor (FGF), and transforming growth factor-β (TGF-β), by binding to their receptor, stimulate cell proliferation and extracellular matrix synthesis, among other functions. EGF in rat calvariae cultures has been reported to stimulate DNA synthesis and increase osteoblast proliferation.

Bone tissue is made up of minerals such as hydroxyapatite and ions that are necessary for the mineralization of the organic bone matrix; 90% of the matrix is made up of type I collagen, as well as water molecules, bone-forming cells (osteoblasts) and cells that break down bone (osteoclasts); these cells regulate the balance between bone formation and degradation^15^.

It is known that during tissue repair, ascorbic acid (AA) is important for collagen synthesis as it acts as a reducing cofactor of ions in the hydroxylation of proline and lysine, which is essential for the crosslinking and stability of the triple helix of collagen, which is subsequently mineralized by depositing hydroxyapatite crystals. AA is essential for bone formation during osteoblast differentiation and proliferation^8^^,^ 16 ^–^ 19. AA deficiency inhibits collagen synthesis. *In vitro*, AA promotes osteoblast differentiation and activates transcription factors, thus influencing bone matrix gene expression^15^^,^ 20.

The objective of this work was to evaluate the effect of AA and EGF on the repair of a noncritical bone defect by means of the biomechanical analysis of destructive three-point bending tests.

## Methods

### Animals

All animal procedures were performed according to the National Rehabilitation Institute LGII, Guide for Care and Use of Laboratory Animals, compliant with the National Institutes of Health (NIH, USA) Guide for Care and Use of Laboratory Animals. Twenty-four male Wistar rats weighing 300 ± 50 g with dark-light cycles of 12:12, they were fed with a commercial balanced diet and purified water *ad libitum.*

### Groups

**Figure 1:**
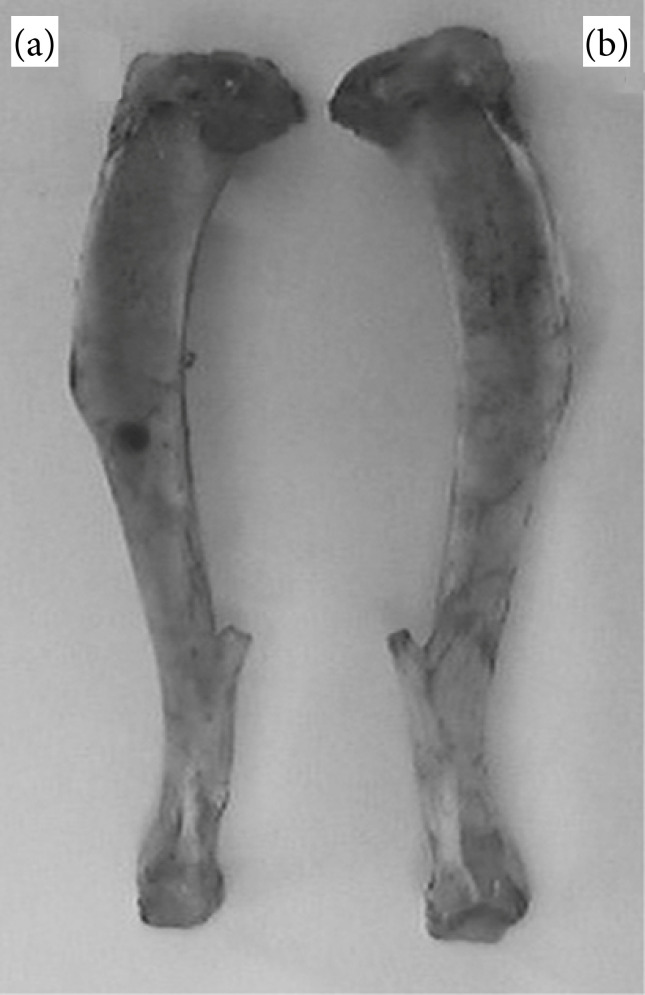
Bone defect sample tibiae. **(a)** Right tibia showing the non-critical bone defect; **(b)** Left intact, control, tibia.

The animals were randomly divided into four groups of six rats each and were organized as follows: G-1, control rats intact with a weight proportional to that of the other groups; G-2, rats with a tibia defect with application of AA, in a single dose on the day of surgery; G-3, rats with a tibia defect with application of EGF in a single dose on the day of surgery; and G-4, rats with a tibia defect with application of AA and EGF in a single dose on the day of surgery. The rats were left to recover for 3 weeks. In all groups, tibias were tested to the contralateral side (Fig. 1). The smallest possible number of rats was used to carry out this study.

### Ascorbic acid (AA) and EGF

Five μg/5μL of AA (Biocheika); or, 100 ng/5 μL solution of murine EGF (GIBCO); or, 5 μg/5μL AA plus 100 ng/5μL EGF was administered appropriately.

### Bone defect and biomechanical test

A noncritical bone defect was made in the right tibia of all animals^21^. General anesthesia was induced by 50 mg/kg intraperitoneal sodium pentobarbital and supplemented as needed (anesthesia plane was monitored by a paw withdrawal reflex). The experimental member was shaved and washed with iodopovidone (8 g/100 mL, Dermodine, DEGASA). A 1-cm incision was made on the tibial crest, taking care not to damage the underlying bone or the adjacent muscle. The superficial fascia was separated from the skin and the tibia was exposed. A 1-mm diameter unicortical defect was made in the region of interest using an electric drill (Mini drill Pros Kit Model PK-500) with a ball-shaped tungsten burr for bone surgery, then the experimental treatment was placed into it and the wound was closed with separated sutures (000 Atramat surgical silk, Mexico). The animals were evaluated every third day, verifying their general health status and the experimental limb. After 3 weeks the rats were sacrificed in a CO_2_ chamber. Biomechanical tests were performed on a universal testing machine (Instron 4502, Instron Inc., Canton, MA, USA) according to Bak et al.^22^. This time window of analysis was chosen because it has been demonstrated through histopathological, histochemical, and morphometric studies that, after 3 weeks, a noncritical bone defect is filled with new bone, but not repaired^9^^,^ 10. Furthermore, there is scanning electron microscopy evidence^21^ that within 14 days a fracture in the rat tibia is not yet repaired, the bone callus is observed but is subsequently calcified at 30 days.

**Figure 2:**
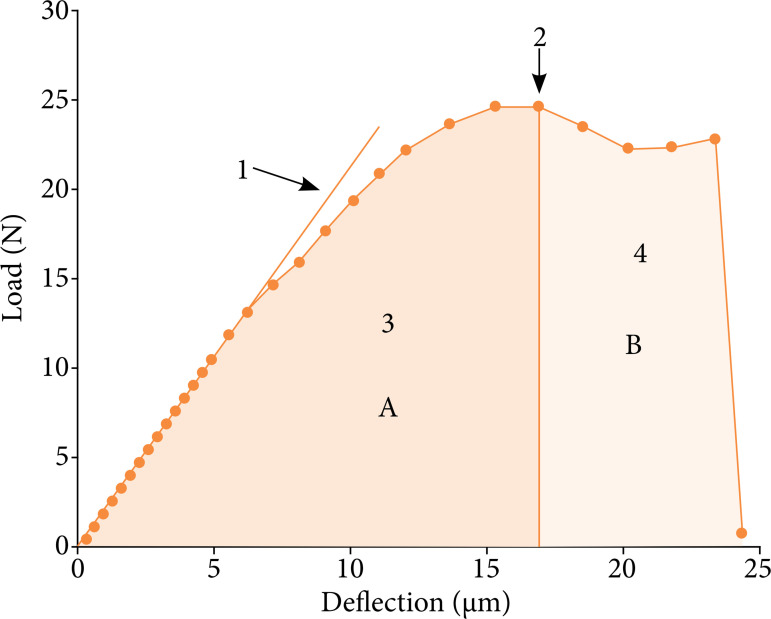
Measurement of biomechanical parameters. (1) Rigidity: slope of the load-displacement curve in its initial linear portion; (2) Resistance: maximum load recorded, highest point of the graph; (3) Energy at maximum load: area A under the curve, it is the energy so that the tibia reaches its point of greatest resistance; (4) Maximum energy: total area under the curve (area A + area B), i.e., total energy to failure.

The data obtained from load displacement were recorded through the interface of the universal testing machine and captured with a conventional personal computer (PC). The load displacement graphs were made with the data obtained during the tests and from these graphs by means of the Origin 8 program (Origin Lab, MA, USA), stiffness, strength, energy at maximum load and maximum energy (Fig. 2) were calculated. For each group, measurements were normalized to the control tibia from each animal.

### Statistical Analysis

Based on the sample size and assuming non-normality of the data, statistical analysis was performed with the nonparametric Mann–Whitney U test to compare between groups^23^. The level of significance was p < 0.05. Analysis was performed with the software SPSS 17.0 (SPSS Inc., Chicago, IL, USA).

## Results

The weight of the rats at the beginning of the experiment was between 300 ± 50 g of body weight (bw); at sacrifice after 3 weeks the bw was 400 ± 30 g, having a gain between 100 ± 30 g, reflecting normal growth for their age and strain. Throughout the experiment, all rats were in good health and supported the experimental limb, fed, and drank water on demand. From the biomechanical trial, of the three groups, 21.7% of the fractures were short obliques and 78.2% were transverse at the site of the defect. The average length for the tibia was 41.4 ± 1.2 mm.

**Figure 3:**
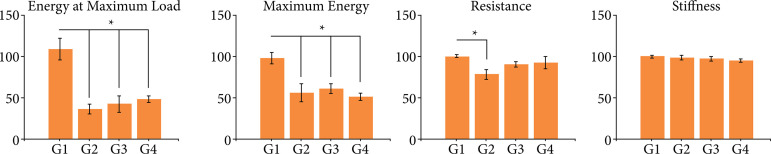
Biomechanical parameters analyzed between groups (values are mean ± standard error of the mean); *p < 0.5.

In the results of the Mann–Whitney U test (Table 1 and Fig. 3), when comparing groups G-1 vs. G-2 significant differences were found only for stiffness, and when comparing groups G-1 vs. G-3 no significant differences were found for strength and resistance; Similarly, for groups G-1 vs. G-4, no significant difference was found for strength and stiffness.

**Table 1:** Results of the Mann–Whitney U test.

	Energy at Maximum Load (N-mm)	Maximum Energy (N-mm)	Resistance (N)	Stiffness (N/mm)
G-1 vs. G-2	0.004*	0.010*	0.006*	0.749
G-1 vs. G-3	0.006*	0.006*	0.055	0.423
G-1 vs. G-4	0.004*	0.004*	0.423	0.150

* Statistically significant difference p < 0.05.

## Discussion

Advances in the treatment of bone defect repair are of importance for orthopedic, dental, and maxillofacial surgery, since, by reducing the time spent on recovery, costs for the patient and the health system are reduced.

In this study, either a single dose of AA (G-2), a single dose of EGF (G-3) or a single dose of AA plus EGF (G-4) was applied locally, with the aim of stimulating the process of bone repair in the rat tibia noncritical bone defect model in which defect repair was assessed by destructive three-point bending test biomechanical analysis. The noncritical bone defect model reported by our group can be used to study various bone defect repair strategies^24^.

For groups G-1 vs G-3 and G-1 vs G-4 at 3 weeks after surgery, no significant differences were found for strength and stiffness, which indicate that both groups recovered two of the biomechanical properties of an intact tibia: the total energy and energy at maximum load. Observing these results, the EGF that was applied in both groups is the one that is acting on bone repair, not the AA, since combining it with EGF has a similar result as EGF alone.

It has been reported that EGF acts in the early phase of repair, when it promotes and regulates the proliferation of osteoblasts; it is also reported that in the late phase of repair EGF is involved in bone differentiation and mineralization^25^^,^ 26, which agrees with our results for both groups treated with EGF, and, although they did not recover all the biomechanical parameters studied, strength and stiffness were recovered.

Bilal et al.^26^ reported higher radiographic score and higher levels of bone morphogenetic protein in a segmental defect of the femur treated with EGF alone and with a combination with plasma rich in platelets; in addition, EGF has benefits in orthopedic surgery, bone repair, diabetic foot ulcer surgery, and experimental dentistry. These results agree with what is reported here regarding the benefit of EGF in bone repair.

Likewise, it has been reported that EGF can be effective in bone regeneration in orthodontics. In bone marrow mesenchymal stromal cell cultures, in dental pulp mesenchymal cells, EGF enhances mineralization and participates in osteogenic differentiation^27^.

On the other hand, antagonistic effects have been reported with respect to the action of EGF on bone tissue. Some studies report that EGF contributes to bone synthesis, while others have reported that EGF contributes to bone resorption^2^^,^ 14. This antagonistic effect of EGF may explain the results reported here; however, other aspects could be considered to explain the differences with our results such as the different strategies used, the different concentrations, the variability of the EGF doses or the experimental model used.

For G-1 versus G-2, three weeks after surgery, no significant differences in stiffness were found, indicating that only the stiffness is equal to that of an intact tibia.

Although AA has been reported to favor collagen synthesis, the synthesis of this protein is a complex process, as are its post-translational modifications. Aghajanian in 2015^20^, points out that knowing the detailed molecular signaling cascades of AA that act on bone cells could complement the therapeutic strategies used and even propose new treatments^20^. On the other hand, Weinstein in 2001^28^, mention that, in young populations, the lack of AA in the diet affects the formation of the bone matrix, which leads to bone fragility and later to fracture^28^ in addition to affecting the activity of the immune system^20^.

Regarding studies on human bone with AA, Aghajanian et al. in 2015^20^ mention that inconsistencies have been reported, perhaps due to the variability of methodologies and experimental designs. This agrees with what was observed in this work. Despite the existence of several reports where AA benefits bone repair, we did not find it beneficial from the biomechanical point of view.

Both AA and EGF have antagonistic effects on bone tissue, as they can accelerate osteoclast formation and osteoclast death. AA promotes osteoclastogenesis in cultures containing both osteoclasts and osteoblasts. This could partly explain our results; however, more studies are required to elucidate the specific pathways in which AA results in a beneficial effect on bone repair.

There are studies of AA as an antioxidant in animal models, where they found a reduction in bone loss, although not enough for total bone recovery. Either in *in vitro* studies where the effect of AA on collagen production is known and osteoblast-specific genes are expressed; and *in vivo*, to specifically guide the pathways that lead to recovery from a fracture or bone defect^16^^,^ 17 ^,^ 20.

On the other hand, it has been reported that, depending on the concentration, growth factors show a heterogeneous effect, and even a totally opposite effect^27^. From our results here and to continue with this work, we propose to evaluate different concentrations of EGF to find the most suitable to repair a bone defect and to induce the recovery of all biomechanical properties.

## Conclusion

EGF and the combination of AA-EGF applied to a noncritical bone defect in the rat tibia favors the recovery of the biomechanical parameter’s strength and stiffness, three weeks after surgery.

## Data Availability

All datasets were generated and analyzed in the current study.
